# Early troponin I in critical illness and its association with hospital mortality: a cohort study

**DOI:** 10.1186/s13054-017-1800-4

**Published:** 2017-08-16

**Authors:** Annemarie B. Docherty, Malcolm Sim, Joao Oliveira, Michael Adlam, Marlies Ostermann, Timothy S. Walsh, John Kinsella, Nazir I. Lone

**Affiliations:** 1 Department of Anaesthesia, Critical Care and Pain Medicine, University of Edinburgh, Royal Infirmary Edinburgh, 2nd Floor Anaesthetics Corridor, Edinburgh, EH16 4SA UK; 2 Centre for Inflammation Research, University of Edinburgh, Edinburgh, UK; 3 Academic Unit of Anaesthesia, Pain & Critical Care, University of Glasgow, Glasgow, UK; 4 Department of Critical Care, King’s College London, Guys and St Thomas’ Hospital, London, UK; 5 Internal Medicine Department, Hospital Jose Joaquim Fernandes, Beja, Portugal; 6 Usher Institute of Population Health Sciences and Informatics, University of Edinburgh, Edinburgh, UK

**Keywords:** Troponin, Critical care, Hospital mortality

## Abstract

**Background:**

Troponin I (TnI) is frequently elevated in critical illness, but its interpretation is unclear. Our primary objectives in this study were to evaluate whether TnI is associated with hospital mortality and if this association persists after adjusting for potential confounders. We also aimed to ascertain whether addition of TnI to the Acute Physiological and Chronic Health Evaluation II (APACHE II) risk prediction model improves its performance in general intensive care unit (ICU) populations.

**Methods:**

We performed an observational cohort study with independent derivation and validation cohorts in two general level 3 ICU departments in the United Kingdom. The derivation cohort was a 4.5-year cohort (2010–2014) of general ICU index admissions (*n* = 1349). The validation cohort was used for secondary analysis of a prospective study dataset (2010) (*n* = 145). The primary exposure was plasma TnI concentration taken within 24 h of ICU admission. The primary outcome was hospital mortality. We performed multivariate regression, adjusting for components of the APACHE II model. We derived the risk prediction score from the multivariable model with TnI.

**Results:**

Hospital mortality was 37.3% (*n* = 242) for patients with detectable TnI, compared with 14.6% (*n* = 102) for patients without detectable TnI. There was a significant univariate association between TnI and hospital mortality (OR per doubling TnI 1.16, 95% CI 1.13–1.20, *p* < 0.001). This persisted after adjustment for APACHE II model components (TnI OR 1.05, 95% CI 1.01–1.09, *p* = 0.003). TnI correlated most strongly with the acute physiology score (APS) component of APACHE II (*r* = 0.39). Addition of TnI to the APACHE II model did not improve discrimination (APACHE II concordance statistic [c-index] 0.835, 95% CI 0.811–0.858; APACHE II + TnI c-index 0.837, 95% CI 0.813–0.860; *p* = 0.330) or other measures of model performance.

**Conclusions:**

TnI is an independent predictor of hospital mortality and correlates most highly with the APS component of APACHE II. It does not improve risk prediction. We would not advocate the adoption of routine troponin analysis on admission to ICU, and we recommend that troponin be measured only if clinically indicated.

**Electronic supplementary material:**

The online version of this article (doi:10.1186/s13054-017-1800-4) contains supplementary material, which is available to authorized users.

## Background

Troponin is used routinely in combination with clinical signs, symptoms and electrocardiography to diagnose or rule out myocardial infarction (MI) [1], and also as a prognostic marker in pulmonary embolism [2]. In critical illness, troponin is frequently elevated, but its interpretation in this context is uncertain. Researchers in several studies of intensive care unit (ICU) and high-risk surgical populations have described significant crude associations between troponin elevation and increased hospital and longer-term mortality [3–5]. There has been increasing interest in the use of troponin in risk stratification in major non-cardiac surgery. In the Vascular Events in Noncardiac Surgery Patients Cohort Evaluation (VISION) study, investigators recently found that peak post-operative troponin T (TnT) was significantly associated with 30-day mortality after adjustment for patient characteristics, and type of surgery [6]. However, the mechanism of a causal association between troponin elevation and mortality is uncertain. Specifically, the aetiology of troponin release is likely multifactorial, and the relative importance of inflammatory and ischaemic myocardial injury is uncertain. In addition, there is conflicting evidence regarding whether troponin is an independent predictor of hospital mortality after adjustment or stratification for severity of illness in critically ill patients [7–10].

The use of multivariable risk prediction models which include diagnosis, severity of illness and other patient characteristics is standard in intensive care and enables benchmarking of practice and outcomes across units [11]. The Scottish Intensive Care Society Audit Group (SICSAG) uses the Acute Physiological and Chronic Health Evaluation II (APACHE II) model [12], which combines acute physiological derangement and presenting diagnosis with chronic health status and age. It has been validated and recalibrated using Scottish data [13]. Addition of new variables may improve the accuracy of existing models; for example, the UK Intensive Care National Audit and Research Centre recently improved the accuracy and discrimination of its model by including lactate [14]. The strong univariate association of troponin with mortality makes it an ideal candidate predictor variable to assess in current risk prediction models.

In the present study, we aimed to explore the possible independent relationship between early troponin I (TnI) and hospital mortality in ICU patients. We explored univariate associations between early TnI concentration and mortality. We then explored how this relationship was modified by adjustment for potential confounding variables that predict death during the first 24 h of ICU admission. Finally, we investigated whether the addition of TnI to the existing APACHE II risk prediction model improves its performance as a predictor of hospital mortality in general critical care populations.

## Methods

### Data sources and participants

Two prospectively collected datasets were available with routine TnI measurement in ICU patients from two distinct ICUs. Glasgow Royal Infirmary (GRI), Scotland, collected TnI samples on all patients admitted to ICU three times each week (on Mondays, Wednesdays and Fridays) from 1 January 2010 through 30 June 2014. These measurements were supplemented by clinically indicated TnI measurements at clinician discretion (Glasgow dataset). Ostermann et al. also performed routine TnI measurement (in addition to their published TnT) within 24 h of ICU admission to a large London ICU (St Thomas’ hospital) for all patients recruited to an observational study to investigate the impact of serial troponin measurements on the diagnosis of MI and hospital and 6-month mortality in patients admitted to ICU with non-cardiac diagnoses (London dataset) [3].

For the Glasgow dataset, we linked TnI data; clinical data extracted from the hospital clinical information system (CareVue®; Koninklijke Philips Electronics N.V., Eindhoven, The Netherlands); and routinely collected, administratively linked registry data derived from the SICSAG [15], Scottish Morbidity Record of acute hospital admissions (SMR01) and Scottish death records. All data were anonymised prior to release to the researchers, and therefore an ethics waiver was granted by the local research ethics committee (West of Scotland Research Ethics Service), and approvals from NHS Greater Glasgow and Clyde Caldicott Guardian and SICSAG were obtained.

Methods for the London dataset are described elsewhere [3]. TnI measurements were part of the original protocol and were reported in a subsequent publication [16]. Data were anonymised prior to release to the researchers. We restricted the dataset to patients in whom TnI measurements were taken in the first 24 h of ICU admission. All diagnoses were recorded as free text, which we mapped to APACHE II diagnosis categories.

### Variables

The primary outcome was hospital mortality. The primary predictor was TnI. We entered TnI as a continuous variable. We assessed linearity of TnI using fractional polynomials and identified that a logarithmic transformation of TnI best represented the data. We used a base 2 logarithmic transformation to facilitate clinical interpretation.

Plasma TnI for the Glasgow dataset was measured using the ARCHITECT STAT Sensitive Troponin-I assay (limit of detection 0.01 μg/L, coefficient of variation [CV] 10% at 0.04 μg/L, quoted analytical range 0.00–50.00 μg/L; Abbott Diagnostics, Lake Forest, IL, USA). The assay used for the London dataset was the ADVIA Centaur® TnI*-*Ultra® three-site sandwich immunoassay (Siemens Medical Solutions, Malvern, PA, USA). The quoted analytical range was 0.006–50 μg/L, total CV was 2.7–5.3% (measured between 0.08 and 27.2 μg/L), reference range < 0.039 μg/L. The lower limit for reporting was 0.04 μg/L. The assays remained the same throughout the study periods. We classified TnI < 0.04 μg/L (undetectable) as ‘0 μg/L’; therefore, we added a small constant (log_2_[TnI + 0.001]) to all values. We describe the study cohort stratified by TnI status for ease of clinical interpretation.

### Association of TnI and hospital mortality

We performed univariate logistic regression to assess the unadjusted association of TnI and hospital mortality, and we used multivariable logistic regression to assess the association of TnI and hospital mortality after adjustment for confounders. For multivariable analyses, we adjusted for the APACHE II risk prediction model. To explore potential mechanisms underlying the association between TnI and hospital mortality, we assessed individual components of the APACHE II risk prediction model: age, chronic health points, emergent surgical admission, acute physiology score (APS) and APACHE II diagnosis on ICU admission. We assessed correlation between TnI and components of the APACHE II model using the square root of the *R* ^2^ value (see Additional file 1). We then adjusted for each component separately for the relationship between hospital mortality and TnI, and we compared the impact that each component had on the OR for hospital mortality by TnI. For details regarding missing data, *see* online supplement.

We performed a number of subgroup and sensitivity analyses for the associations between TnI and hospital mortality:

1. For routine vs clinical TnI collection, the Glasgow dataset comprised all TnI samples taken within 24 h of ICU admission. We compared characteristics and outcomes of patients who had a TnI sample taken with routine morning blood laboratory work-up on a Monday, Wednesday or Friday (‘routine’) with those who had a TnI work-up done on Tuesday, Thursday, Saturday or Sunday at clinical discretion (‘clinical’). We also restricted analyses to patients who had routine TnI samples taken to explore any bias that might result from inclusion of clinically indicated TnI samples.
2. We restricted analyses to patients with an APACHE II comorbidity diagnosis of severe cardiac disease (New York Heart Association class IV). We hypothesised that the mechanism of TnI release may be different in patients with severe cardiovascular disease.
3. We included an interaction term for sex because TnI release is higher in men than in women, owing to increased muscle mass of the left ventricle [17].
4. We entered TnI as a binary categorical variable consistent with diagnostic thresholds for this assay: ‘positive’ (above the limit of reporting, ≥ 0.04) vs ‘negative’ (below the limit of reporting, < 0.04).

### Sensitivity analysis

A significant proportion of patients did not have TnI taken within 24 h of admission. For those patients who had samples taken at clinical discretion, we believe that these are ‘missing not at random’, because the mechanism of missingness is likely related to the TnI value. However, for those samples taken as part of routine care, we believe that missing values may fulfil criteria to be ‘missing at random’. We therefore imputed the missing TnI values using multiple imputation by chained equations to assess the robustness of our analysis for this subgroup of patients (*see* online supplement).

### Addition of TnI to APACHE II risk prediction model

To explore whether TnI concentration measured during the first 24 h in ICU added to the risk prediction properties of the APACHE II, we undertook a derivation and validation of the model using the datasets with and without the inclusion of TnI data. For model development, we reconstructed the APACHE II risk prediction model [12] using raw data and derived the predicted mortality empirically from the multivariable model. We constructed age points, chronic health points, emergency surgical admission and APS in the same way as the APACHE II risk prediction model. We modified the APACHE diagnostic codes by recoding diagnostic codes with frequency < 10 into ‘other’ disorder for that system to ensure stability of coefficient estimates. We then added TnI to the APACHE II model using empirically weighted coefficients derived from the model. This ensured that we did not underestimate any potential contribution to the model from TnI [14].

For model validation, we used the whole Glasgow dataset to develop the model and applied coefficients from the development dataset to the London dataset in line with current guidelines [18]. We reported the performance of the risk prediction model for the following: calibration, discrimination, AUROC (concordance statistic [c-index], DeLong’s test for two correlated ROC curves), overall performance score (Brier score) and overall fit (Akaike information criterion and *R* ^2^) [18]. We compared how the performance of the APACHE II risk prediction model was altered by the inclusion of TnI on the first day in ICU as evidence for early contribution to risk of death.

All data were analysed using the R statistical software package (version 3.3.2; R Core Team, Vienna, Austria) [19]. We used the following packages: ggplot2, mfp, rms and pROC [20–23] (R code; *see* online supplement).

## Results

### Participants

**Fig. 1:**
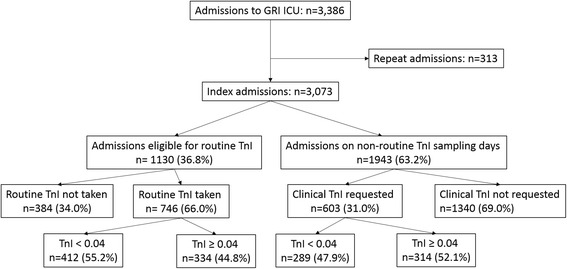
Flow of patients through study. GRI Glasgow Royal Infirmary, ICU Intensive care unit, TnI Troponin I

There were 3073 index admissions to GRI between 1 January 2010 and 30 June 2014 (Fig. 1). A total of 1349 patients (43.9%) had TnI taken within 24 h of admission to ICU (‘Glasgow dataset’). Patients who did not have TnI taken (*n* = 1724, 56.1%) were younger, were more likely to have an elective admission, and had lower APACHE II scores and mortality (ICU, hospital and 6-month) (Additional file 1: Table S1). The mean age of patients who had TnI taken within 24 h was 59.6 years (SD 16.6); 607 (45.0%) were female; and 59.5% were emergency medical admissions (Table 1). A proportion of 80.0% had no severe comorbidity (APACHE II score); the mean APACHE II score was 20.1 (8.1); and median predicted mortality was 31.7%. Patients with positive TnI (≥0.04 μg/L) compared with negative TnI (<0.04 μg/L) were older, and a higher proportion were male, had emergency medical admissions, had higher APACHE II scores, and died (in ICU, in hospital and 6-month) (Table 1). The median TnI in those with a detectable level (*n* = 648) was 0.23 μg/L (Q1 0.09, Q3 0.97, maximum 69.91).

**Table 1:** Baseline characteristics

	Overall	%	TnI − ve	%	TnI + ve	%	*p* Value
	(*n* = 1349)		(*n* = 701)		(*n* = 648)		
Age, years, mean (SD)	59.6	16.6	56.7	16.8	62.8	15.8	< 0.001
Female sex	607	45.0	337	48.1	270	41.7	0.021
Admission type							< 0.001
Elective surgery	333	24.7	198	28.2	135	20.8	
Emergency surgery	213	15.8	172	24.5	41	6.3	
Emergency medical	803	59.5	331	47.2	472	72.8	
Deprivation quintile (*n* = 1143)							0.081
1 (most deprived)	622	46.1	298	42.5	324	50.0	
2	193	14.3	108	15.4	85	13.1	
3	94	7.0	52	7.4	42	6.5	
4	123	9.1	66	9.4	57	8.8	
5 (least deprived)	111	8.2	68	9.7	43	6.6	
APACHE comorbidities							0.030
0	1079	80.0	589	84.0	490	75.6	
1	191	14.2	83	11.7	109	16.8	
≥ 2	79	5.9	29	4.1	49	7.6	
TnI, μg/L, median [IQR], maximum	0.0	[0.00–0.21], 69.91	–	–	0.23	[0.09–0.96], 69.91	
Outcomes							
ICU mortality	292	21.6	73	10.4	219	33.8	< 0.001
Hospital mortality	344	25.5	102	14.6	242	37.3	< 0.001
6 month mortality	419	31.1	142	20.3	277	42.7	< 0.001
APACHE II predicted mortality, %, median [IQR]	25.3	[9.7–49.7]	17.2	[6.9–32.2]	40.4	[19.8–63.1]	< 0.001
APACHE II score, mean (SD)	20.1	8.1	16.9	6.6	23.6	8.1	< 0.001
ICU LOS, median [IQR]	2.9	[1.3–6.8]	2.7	[1.1–6.6]	3.2	[1.6–7.7]	< 0.001

*Abbreviations: APACHE II* Acute Physiological and Chronic Health Evaluation II, *ICU* Intensive care unit, *LOS* Length of stay, *TnI* Troponin I

Stratified by overall population. TnI –ve (<0.04 μg/L) vs TnI + ve (≥0.04 μg/L). *p* value: test between TnI –ve and TnI + ve: chi-square test/test for trend for categorical variables, *t* test for parametric continuous variables, Mann-Whitney *U* test for non-parametric variables. There are no missing data in this table’ patients with missing TnI values are discussed in the online supplement

### Association of TnI and hospital mortality

**Fig. 2:**
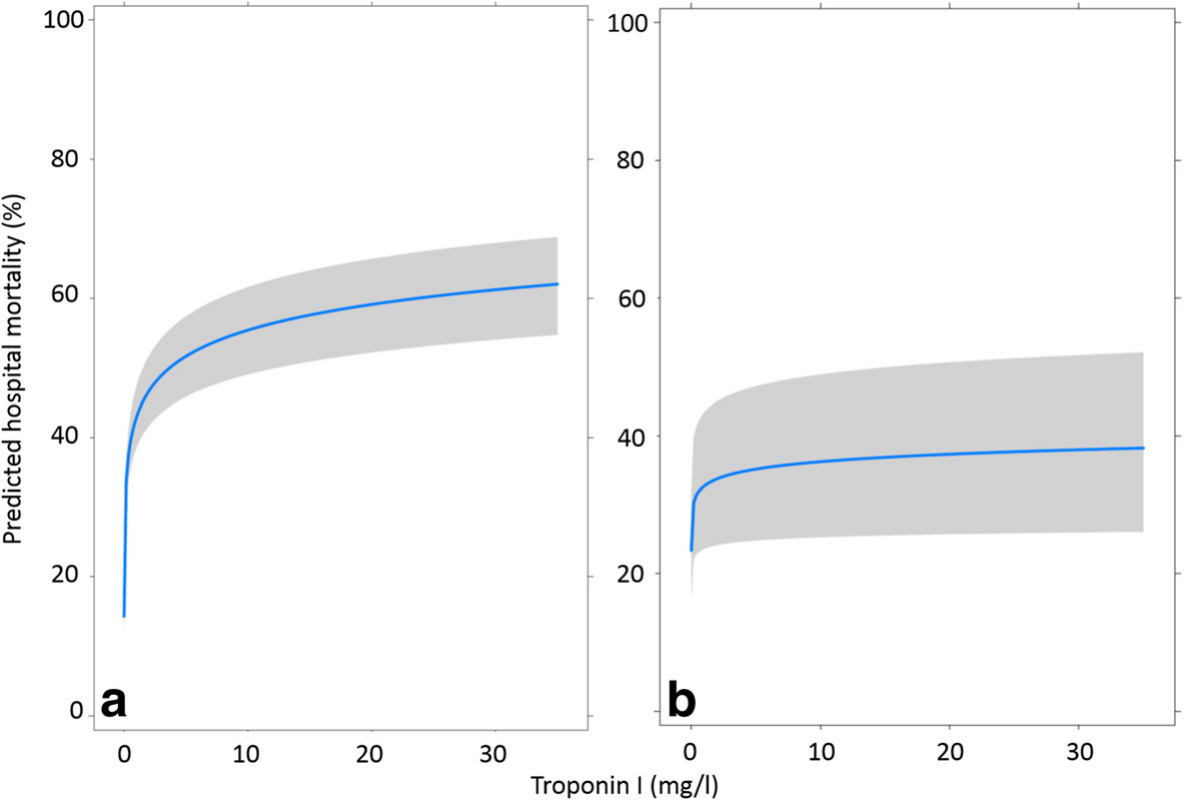
Association between troponin I (TnI; in μg/L) and hospital mortality. a Univariate association (OR per doubling of TnI 1.16, 95% CI 1.13–1.20, p < 0.001). b Multivariate association between TnI and hospital mortality once added to the Acute Physiological and Chronic Health Evaluation II model (OR 1.05, 95% CI 1.01–1.09, p < 0.001)

Hospital mortality was 37.3% (*n* = 242) for TnI-positive patients compared with 14.6% (*n* = 102) for TnI-negative patients (Table 1). There was a significant univariate association between TnI as a continuous term and hospital mortality (Fig. 2a) (OR per doubling of TnI 1.16, 95% CI 1.13–1.20, *p* < 0.001). This is equivalent to an increase in predicted hospital mortality from 27.2% to 30.3% if TnI increases from 0.04 μg/L to 0.08 μg/L and from 42.9% to 46.7% for an increase in TnI from 1.0 μg/L to 2.0 μg/L. After adjustment for the APACHE II risk prediction model, TnI remained an independent predictor of hospital mortality, but the magnitude of prediction was reduced (Fig. 2b) (OR 1.05, 95% CI 1.01–1.09, *p* = 0.003).

### Correlation of TnI with components of APACHE II model

TnI was most highly correlated with the APS (*r* = 0.39) and diagnostic category (*r* = 0.40) components of the APACHE II model (Table 2). There was some correlation with age points (*r* = 0.17) but minimal correlation with chronic health points (*r* = 0.11) or emergency surgery (*r* = 0.10). This was reflected by the impact that each component had on the association between TnI and hospital mortality. APS had the greatest impact, reducing the odds of hospital mortality from 1.16 (95% CI 1.13–1.20) to 1.08 (95% CI 1.05–1.11).

**Table 2:** Correlation of Acute Physiological and Chronic Health Evaluation II with troponin I

Component of APACHE II	Pearson coefficient (*r*)	TnI OR for hospital mortality^a^	95% CI
Unadjusted TnI	–	1.16	1.13–1.20
Age points	0.17	1.15	1.12–1.19
Chronic health points and emergency surgery	0.11	1.16	1.13–1.19
APS	0.39	1.08	1.05–1.11
Emergency surgery	0.10	1.16	1.12–1.19
Diagnostic category	0.40	1.11	1.07–1.15

*Abbreviations: APACHE II* Acute Physiological and Chronic Health Evaluation II, *APS* Acute physiology score component of Acute Physiological and Chronic Health Evaluation II, *TnI* Troponin I

Initially, each component was separately added to a univariate analysis with log_2_(TnI + 0.001) as the dependent variable. The Pearson coefficient (r) is the square root of the R^2^ (coefficient of determination) and assesses the correlation between the two variables

^a^We adjusted for each component separately for the relationship between hospital mortality and TnI, and we compared the impact that each component had on the OR for hospital mortality by TnI. Unadjusted OR per doubling TnI: 1.16 (95% CI 1.13–1.20, *p* < 0.001)

### Addition of TnI to APACHE II risk prediction model

#### Derivation

**Fig. 3:**
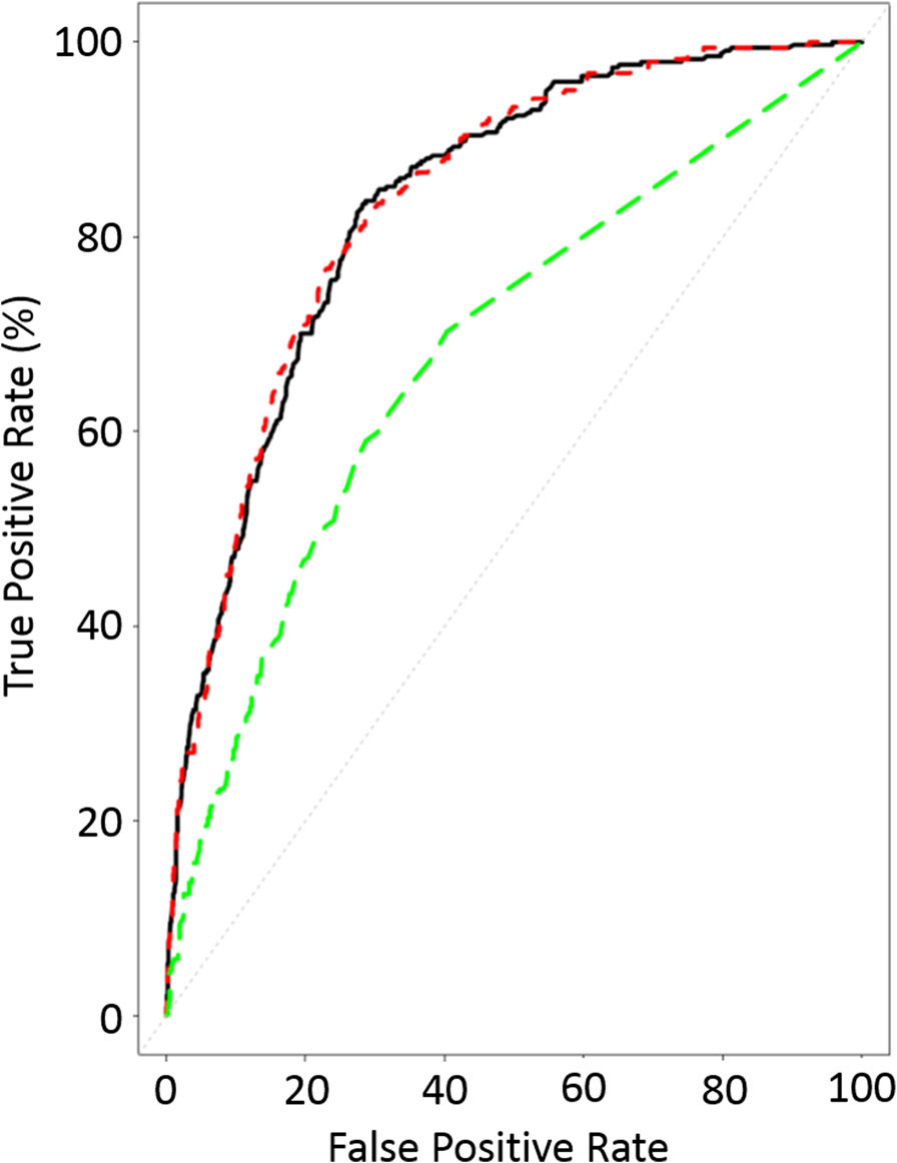
ROC curves: Acute Physiological and Chronic Health Evaluation (APACHE) (*black solid line*; concordance statistic [c-index] 0.847), APACHE + troponin (*red dashed line*; c-index 0.846) and troponin I (*green dashed line*; c-index 0.696)

The variables comprising the APACHE II model applied to this dataset resulted in a c-index of 0.835 (95% CI 0.811–0.858) (Fig. 3), demonstrating good discriminatory ability and calibration (Additional file 1: Figure S1). The addition of TnI as a logarithmic term did not improve the discriminatory power of the model (*p* = 0.330, c-index 0.837, 95% CI 0.813–0.860) (Fig. 3), nor did it improve other assessments of model performance (Table 3, Additional file 1: Figure S2).

**Table 3:** Model comparison for Glasgow and London datasets (derivation), Acute Physiological and Chronic Health Evaluation II vs Acute Physiological and Chronic Health Evaluation II + troponin I

	Glasgow		London	
	APACHE	APACHE + troponin	APACHE	APACHE + troponin
AUC (95% CI)	0.823 (0.811–0.858)	0.826 (0.813–0.860)	0.737 (0.630–0.845)	0.752 (0.645–0.859)
AIC	1198.0	1188.8	144.5	143.0
*R* ^2^	0.340	0.349	0.330	0.355
Brier score	0.141	0.140	0.120	0.118
*p* Value		0.331		0.010

*AIC* Akaike information criterion, *APACHE* Acute Physiological and Chronic Health Evaluation

*p* value: DeLong’s test for two correlated ROC curves. Glasgow model coefficients applied to London dataset (validation)

#### Validation

There were 145 patients in the London dataset, which had demographics similar to the Glasgow dataset (Table 1, Additional file 1: Table S3). There was a high proportion of emergency medical admissions (71.0%), with a mean APACHE II score of 19.1. Hospital mortality was 20.0%. TnI was significantly associated with hospital mortality (unadjusted OR TnI 1.23, 95% CI 1.09–1.41). This association was attenuated after adjustment for potential confounders (OR 1.16, 95% CI 0.99–1.36).

Applying coefficients derived from the Glasgow data-derived model, we found a small but statistically significant improvement in the c-index after the addition of TnI; however, CIs were wide (standard APACHE II model c-index 0.735, 95% CI 0.631–0.845; APACHE II + TnI c-index 0.752, 95% CI 0.645–0.859; *p* = 0.010). Other assessments of model performance showed no improvement with the addition of TnI (Table 3).

### Subgroup and sensitivity analyses: multivariate association of TnI and hospital mortality

**Fig. 4:**
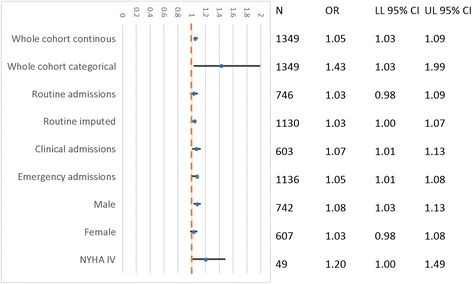
Sensitivity and subgroup analyses. OR of hospital mortality for doubling of TnI as a continuous variable for different subgroups after adjusting for the Acute Physiological and Chronic Health Evaluation II (APACHE II) risk prediction model. ‘Whole cohort categorical’ refers to TnI entered as a binary variable: TnI + ve vs TnI − ve using the threshold of the limits of detection (0.04). LL Lower limit of 95% CI, UL Upper limit of 95% CI, NYHA New York Heart Association cardiac disease class IV (APACHE II classification for severe cardiac disease)

Those with a routine TnI sample accounted for 55.3% of samples taken (*n* = 746). Those with a TnI sample taken at clinical discretion accounted for 44.7% (*n* = 603) (Additional file 1: Table S1). Clinical group patients were more likely to be emergency admissions, to have a positive TnI, to have higher APACHE II scores and predicted mortality. ICU length of stay, ICU mortality, and hospital or 6-month mortality were similar. Restriction to routine group patients did not alter the magnitude of association of TnI in the multivariate model, but the association was no longer statistically significant (OR 1.03, 95% CI 0.98–1.09, *p* = 0.287) (Fig. 4). The OR for TnI in our sensitivity analysis where we imputed the missing TnI values in the routine group was 1.04 (95% CI 1.00–1.08, *p* = 0.020) (Fig. 2, Additional file 1: Table S2).

TnI was a significant predictor of hospital mortality when the dataset was restricted to patients (*n* = 49) with a diagnosis of severe cardiac disease (OR 1.20, 95% CI 1.00–1.49) (Fig. 4). The odds of hospital mortality were greater for men (OR 1.08, 95% CI 1.03–1.13) than for women (OR 1.03, 95% CI 0.98–1.08) (Fig. 4), but this difference was not significant when tested for interaction between TnI and sex (*p* = 0.181) (Additional file 1: Figure S2). When TnI was entered as a binary variable ‘positive’ (*n* = 648) vs ‘negative’ (*n* = 701), the OR of hospital mortality for positive TnI was 1.43 (95% CI 1.03–1.99) (Fig. 4).

## Discussion

In this cohort study of two independent datasets, we found that an early raised TnI level was associated with increased mortality in general ICU populations. The magnitude of the association remained significant but was markedly attenuated after controlling for other important predictors of hospital mortality. The addition of TnI to the APACHE II risk prediction model did not result in a clinically relevant improvement of model performance.

Our study has a number of strengths. We had access to routinely measured TnI in a large, well-defined population in the derivation cohort, as well as in a high-quality validation cohort from a prospective study in a different population and setting. This contrasts with previous ICU studies in which researchers have analysed TnI samples taken for clinical purposes [7, 10]. This reduced the risk of bias by clinical indication. Although there have been several large peri-operative studies, this is the largest study to date to investigate troponin in ICU patients, allowing subgroup analyses to assess the robustness of our findings. We used multiple imputation for missing TnI values in patients who were eligible for routine TnI sampling. We also used TnI as both a continuous and a categorical variable. We reported our risk prediction model to current international best practice standards, including externally validating the model rather than internally validating on a proportion of the derivation cohort [18]. Internally validated prediction models make use of data only in the development model and may have an artificially high performance. The external validation dataset provided the heterogeneity that would be encountered in the real-life application of the model [14].

Our study has some limitations. Patients who did not have TnI taken within 24 h of admission to ICU had differences from those who did, which may affect the generalisability of our findings. This could have occurred because ICU case mix can differ between certain weekdays and weekends [24, 25] and because patients who were less sick did not have blood samples taken. Patients who were eligible for routine TnI sampling but did not receive it were younger, were less sick and had much shorter ICU stays. Troponin was reported using the assays in clinical practice during the study period. The assays used in the Glasgow and London studies were different, which may have affected interpretation of the London dataset. Our lower limit of reporting was 0.04 μg/L, which may have resulted in some samples being classified as ‘0’ μg/L, when TnI may have been detectable using a highly sensitive assay, and this may have affected women more [17]. We found no significant interaction between TnI and sex, although our study may be underpowered for this.

A further limitation was the restriction of troponin data to the first 24 h of ICU care. This time point is defined by hospital location rather than by illness stage, and it is therefore potentially subject to lead time bias. For troponin, this could be particularly relevant, given the important association between time of symptom onset and troponin measurement for maximum diagnostic value in acute coronary syndrome. This cannot be controlled in ICU populations, but it was a potential source of variation. We restricted our analyses to the first TnI taken, but assessing the dynamics of TnI release during critical illness may have greater prognostic value. In the future, researchers could explore the dynamics of troponin release in critically ill patients, which might have a stronger association with mortality.

The significant univariate association between TnI and hospital mortality confirms that found in other studies undertaken in critically ill and high-risk surgical populations [3–5, 7–9, 26]. In non-cardiac surgical patients, TnT has also been found to have a significant independent association with hospital and longer-term mortality [5, 26]. Both studies made adjustment for patient characteristics and type of surgery, but they did not include any measure of acute physiological derangement. In critically ill patients, Wu et al. found a significant association between TnI and 6-month mortality after stratifying by high/low APACHE II scores [8]. Babuin et al. found that TnT remained a significant predictor of mortality after adjustment for APACHE III [7]. This was a retrospective study of patients with a high proportion of cardiac disease who had TnT measurements taken for clinical indications. This is consistent with our finding that, in patients with chronic heart disease, TnI remained a predictor of mortality after adjustment.

Our finding that the association between TnI and mortality was significant but markedly attenuated after adjusting for potential confounders suggests that myocardial injury may be on the causal pathway between illness severity and death: the sicker the patient, the more troponin is released, and the higher the risk of death. The causal mechanism of TnI release is unclear. Troponin release has traditionally been associated with myocardial necrosis and forms a key part of the diagnosis of MI [1]. However, there is also increasing evidence that troponin release may occur during myocardial ischaemia in the absence of myocardial necrosis, potentially due to shedding of cytosolic troponin [27]. The physiological stress associated with critical illness may exacerbate oxygen supply-demand imbalance (type II MI) [1, 28], particularly in those with underlying critical coronary artery disease. In sepsis, there are profound haemodynamic alterations in the microcirculation which may lead to alterations in oxygen extraction and tissue oxygenation [29]. In a minority of critically ill patients, troponin release may be caused by increased thrombogenicity, leading to coronary plaque rupture and thrombosis (type I MI). Proposed non-cardiac mechanisms of troponin release in the critically ill include direct toxicity from cytokine release, such as tumour necrosis factor-α and interleukin-6 [30], stretch-mediated troponin release [30], or ongoing subclinical myocardial injury due to uraemia and impaired excretion [31]. Our datasets did not allow us to explore in detail underlying mechanisms of troponin release. However, the APS component represents acute physiological stress and comprises surrogate markers of supply (hypoxia, hypotension, anaemia) vs demand (increased metabolic rate) imbalance, and our finding that TnI is most strongly correlated with the APS component of the APACHE II model supports the hypothesis that myocardial injury may be due to oxygen supply-demand imbalance. Using Bradford-Hill criteria for assessing causal relationships, we showed that there was a biological gradient whereby a greater TnI was associated with increased mortality, and that there was consistency across the literature. However, there was no TnI measurement preceding critical illness, meaning that we were unable to comment on the temporal association. Furthermore, the strength of association between TnI and mortality after adjustment for known confounders was weak, and the mechanism of the association was unclear. This was an observational study, and unmeasured confounders prevent the inference of causality. Further exploration would require propensity or mediation analyses.

## Conclusions

We found that the significant association between TnI and hospital mortality was substantially attenuated after controlling for potential confounders, particularly acute physiological derangement. However, the addition of TnI to the existing APACHE II model did not improve its performance. Given its relative expense, we would not advocate the adoption of routine troponin analysis for general ICU patients on their admission to critical care, and we would recommend that troponin be measured only if clinically indicated. Future studies using higher-generation highly sensitive troponin assays, potentially exploring changes over time, may further inform the relationship between TnI and mortality.

## Additional file

Additional file 1: Table S1. Baseline characteristics. **Table S2.** Baseline characteristics for patients eligible for routine TnI within 24 h of ICU admission. **Table S3.** Baseline characteristics: London dataset. **Table S4.** Coefficients and SE for whole dataset. **Figure S1.** Calibration plot for predicted vs actual probabilityof hospital mortality. **Figure S2.** Association of troponin I (in mg/L) with hospital mortality with interaction term for sex. (DOCX 93 kb)

## Abbreviations

AIC: Akaike information criterion
APACHE II: Acute Physiological and Chronic Health Evaluation II
APS: Acute physiology score component of Acute Physiological and Chronic Health Evaluation II
c-index: Concordance statistic
CV: Coefficient of variation
GRI: Glasgow Royal Infirmary
ICU: Intensive care unit
LL: Lower limit
LOS: Length of stay
MI: Myocardial infarction
NYHA: New York Heart Association
SICSAG: Scottish Intensive Care Society Audit Group
SMR01: Scottish Morbidity Record of acute hospital admissions
TnI: Troponin I, UL, Upper limit
TnT: Troponin T
