# Supplementary material for: Early troponin I in critical illness and its association with hospital mortality: a cohort study
Source: Crit Care. 2017 Aug 16;21:216. doi: 10.1186/s13054-017-1800-4 (PMC5559840; doi:10.1186/s13054-017-1800-4)
Supplement: Additional file 1: Table S1. — Baseline characteristics. Table S2. Baseline characteristics for patients eligible for routine TnI within 24 h of ICU admission. Table S3. Baseline characteristics: London dataset. Table S4. Coefficients and SE for whole dataset. Figure S1. Calibration plot for predicted vs actual probabilityof hospital mortality. Figure S2. Association of troponin I (in mg/L) with hospital mortality with interaction term for sex. (DOCX 93 kb) [file 13054_2017_1800_MOESM1_ESM.docx]

# Online supplement TnI Critical Illness

**Methods**

Correlation between TnI and components of the APACHE II model:

We performed univariate linear regression, with log2(TnI+0·001) as the dependent variable, and each component of the APACHE II model separately as the independent variable. We took the square root of the R^2^ value, equivalent to the Pearson correlation coefficient in univariate analyses. This enabled us to include categorical predictors (not possible in simple correlation) as it uses correlation between observed TnI, and TnI predicted by the regression model (the mean TnI for each group). We then adjusted for each component separately for the relationship between hospital mortality and TnI, and compared the impact that each component had on the odds ratio for hospital mortality by TnI.

**Missing data**

We followed the APACHE II model method which assumes that missing physiological values are normal and are therefore allocated a zero score. Missing APACHE II diagnostic codes were classified as “other miscellaneous” in accordance with current SICSAG practice. Other missing values were not imputed and a complete case analysis was undertaken.

We stratified the dataset dependent on whether samples was taken for “routine” or “clinical” indications. For those patients who had samples taken at clinical discretion, we believe that these are “missing not at random”, as the mechanism of missingness is likely related to the TnI value. It is not valid to perform multiple imputation for missing values when the missingness mechanism is “missing not at random.” However, for those samples taken as part of routine care, we believe that missing values may fulfil criteria to be “missing at random.” We therefore imputed the missing TnI values using multiple imputation by chained equations to assess the robustness of our analysis for this subgroup of patients

**Sample size**

We were limited by the sample size of the existing datasets. Regular TnI collection began for the Glasgow dataset in late 2009, and we included all index admissions between 01/01/2010-30/06/2014, who had a TnI sample taken within 24 hours of admission to ICU. The London dataset comprised 145 patients recruited to their study.

**Online Tables**

**Table S1: Baseline characteristics.** Stratified by “Routine TnI”: TnI taken within 24 hours of ICU admission on Mon/Wed/Fri at 08:00; “Clinical TnI”: TnI taken within 24 hours of icu admission on Tues/Thurs/Sat/Sun. “No TnI”: no TnI taken within 24 hours of ICU admission. TnI positive ≥0.04, TnI negative <0.04. LOS: Length of stay

|  | **Routine TnI** | **%** | **Clinical TnI** | **%** | **No TnI** | **%** |
| --- | --- | --- | --- | --- | --- | --- |
| n (%) | 746 | 24.3 | 603 | 19.6 | 1724 | 56.1 |
| age mean (SD) | 58.4 | 17 | 61.2 | 16 | 56.2 | 15.8 |
| female n (%) | 334 | 44.8 | 273 | 45.3 | 858 | 49.8 |
| admission type |  |  |  |  |  |  |
| elective surgery | 135 | 18.1 | 78 | 12.9 | 440 | 25.5 |
| emergency surgery | 187 | 25.1 | 146 | 24.2 | 510 | 29.6 |
| emergency medical | 424 | 56.8 | 379 | 62.9 | 773 | 44.9 |
| deprivation quintile |  |  |  |  |  |  |
| 1 (most deprived) | 329 | 44.1 | 293 | 48.6 | 781 | 45.3 |
| 2 | 112 | 15.0 | 81 | 13.4 | 235 | 13.6 |
| 3 | 49 | 6.6 | 45 | 7.5 | 143 | 8.3 |
| 4 | 77 | 10.3 | 46 | 7.6 | 120 | 7.0 |
| 5 (least deprived) | 64 | 8.6 | 47 | 7.8 | 144 | 8.4 |
| Missing | 115 | 15.4 | 91 | 15.1 | 301 | 17.5 |
| APACHE comorbidity count |  |  |  |  |  |  |
| 0 | 597 | 80.0 | 482 | 79.9 | 1410 | 81.8 |
| 1 | 109 | 14.6 | 82 | 13.6 | 230 | 13.3 |
| 2 | 27 | 3.6 | 23 | 3.8 | 54 | 3.1 |
| ≥3 | 13 | 1.8 | 16 | 2.6 | 29 | 1.7 |
| TnI +ve n(%) | 334 | 44.8 | 314 | 52.1 | - | - |
| APACHE II score mean(SD) | 19.1 | 8.1 | 21.3 | 7.9 | 15.25 | 7.2 |
| APACHE predicted mortality mean (SD) | 29.5 | 24.7 | 34.5 | 36.7 | 21.2 | 20.2 |
| ICU LOS med (IQR) | 2.9 | 1.3, 6.8 | 2.9 | 1.3, 6.9 | 1.1 | 0.7, 2.5 |
| ICU mortality n(%) | 150 | 20.1 | 142 | 23.5 | 202 | 11.7 |
| hosp mortality n(%) | 179 | 24 | 165 | 27.4 | 255 | 14.8 |
| 6m mortality n(%) | 223 | 29.9 | 196 | 32.5 | 321 | 18.6 |

**Table S2: Baseline characteristics for patients eligible for routine TnI within 24 hours of ICU admission.** Stratified by whether TnI was missing or taken. 19 patients in the missing group died or were discharged before 08:00am when morning bloods were taken.

| **Variable** | **TnI missing** | **%** | **TnI taken** | **%** | **P value** |
| --- | --- | --- | --- | --- | --- |
| n (%) | 384 | 34.0 | 746 | 66.0 |  |
| age (mean (sd)) | 55.8 | 17.5 | 58.4 | 16.1 | 0.011 |
| Female n (%) | 200 | 52.2 | 327 | 43.8 | 0.009 |
| Admission Type n (%) | | | | | <0.001 |
| Elective Surgery | 113 | 29.5 | 189 | 25.4 |  |
| Emergency Surgery | 123 | 32.0 | 139 | 18.6 |  |
| Emergency Medical | 148 | 38.5 | 419 | 56.1 |  |
| APACHE comorbidities n (%) | | | | | 0.094 |
| 0 | 328 | 85.4 | 593 | 79.4 |  |
| 1 | 41 | 10.7 | 46 | 14.8 |  |
| ≥2 | 15 | 3.9 | 260 | 5.8 |  |
| Outcomes | | | | | |
| ICU mortality n (%) | 43 | 11.1 | 132 | 17.7 | 0.004 |
| Hospital mortality n (%) | 53 | 13.7 | 158 | 21.2 | 0.003 |
| 6 month mortality n (%) | 62 | 16.2 | 197 | 26.4 | <0.001 |
| APACHE II Score (mean (sd)) | 14.9 | 7.2 | 19.0 | 7.6 | <0.001 |
| % APACHE II predicted mortality med [IQR] | 9.3 | 4.6, 21.3 | 20.8 | 8.6, 40.8 | <0.001 |
| ICU los med [IQR] | 1.0 | 0.7, 2.4 | 3.1 | 1.7, 6.8 | <0.001 |
| ICU LOS<24 hours n (%) | 157 | 40.8 | 83 | 11.1 | <0.001 |

**Table S3: Baseline Characteristics: London dataset.** Stratified by Overall population, TnI –ve (<0.04mg/l) vs TnI +ve (≥0.04mg/l). P-value: test between TnI –ve and TnI +ve: Chi2 test/test for trend for categorical variables, t-test for parametric continuous variables, Mann-Whitney-U test for non-parametric variables. *OR per doubling of TnI.

|  | **n** | **%** | **TnI –ve** | **%** | **TnI +ve** | **%** | **P value** |
| --- | --- | --- | --- | --- | --- | --- | --- |
| N | 145 |  | 54 |  | 91 |  |  |
| Age years mean (SD) | 61.7 | 17.0 | 57.2 | 18.4 | 64.5 | 15.6 | 0.013 |
| Female (%) | 63 | 43.4 | 19 | 35.2% | 43 | 47.3 | 0.213 |
| Admission type  Elective Surgery  Emergency Surgery  Emergency Medical | 7  38  100 | 4.8  26.2  70.0 | 1  13  40 | 1.9  24.1  74.1 | 6  25  60 | 6.6  27.5  65.9 | 0.358 |
| Troponin +ve | 116 | 80 | - | - | - | - | - |
| Troponin med (IQR,max) | 0.06 | 0.02, 0.33 | - | - | 0.20 | 0.06, 0.81 |  |
| APACHE II score mean (SD) | 19.1 | 6.4 | 14.6 | 7.2 | 20.3 | 8.4 | 0.011 |
| Outcomes | | | | | | | |
| ICU mortality | 28 | 19.3 | 1 | 1.9 | 27 | 29.6 | 0.031 |
| Hospital mortality | 29 | 20.0 | 3 | 5.6 | 38 | 41.8 | 0.010 |
| 6 month mortality | 44 | 30.3 | 3 | 5.6 | 41 | 45.1 | 0.017 |
| OR TnI (95% CI) univariate* | 1.23 | 1.09, 1.41 |  |  |  |  |  |
| OR TnI (95% CI) multivariate* | 1.16 | 0.99, 1.36 |  |  |  |  |  |

**Table S4: Coefficients and Standard Error for whole dataset.** Hospital mortality ~ log(TnI+0.001) + APACHE II Score + Emergency surgery + APACHE II Diagnosis.

| **Variable** | **Whole dataset** | |
| --- | --- | --- |
|  | Coefficient | SE |
| Intercept | -4.29 | 0.26 |
| Log(TnI+0.001) | 0.02 | 0.01 |
| APACHE II Score | 0.16 | 0.01 |
| Emergency surgery | 0.43 | 0.23 |
| APACHE II Diagnosis (ref Respiratory Infection) | | |
| Asthma/Allergy | -1.07 | 0.85 |
| Other Respiratory Infection | -0.08 | 0.28 |
| Pulmonary oedema (non-cardiogenic) | -0.23 | 0.46 |
| Aspiration/poisoning/toxic | -0.74 | 0.46 |
| Other cardiovascular disorder | -0.91 | 0.35 |
| Congestive heart failure | -2.30 | 0.90 |
| Sepsis | 0.16 | 0.24 |
| Post cardiac arrest | -0.06 | 0.30 |
| Cardiogenic shock | 0.47 | 0.47 |
| Multiple trauma | -0.49 | 0.58 |
| Head trauma | -1.13 | 0.66 |
| Seizure disorder | -1.61 | 0.44 |
| ICH/SDH/SAH | 1.19 | 0.43 |
| Other neurological disorder | -0.73 | 0.41 |
| Drug overdose | -2.51 | 0.55 |
| Other metabolic/renal disorder | -1.48 | 0.35 |
| GI bleeding | -0.93 | 0.50 |
| Other gastrointestinal disorder | 0.13 | 0.22 |
| Chronic cardiovascular disease | -0.69 | 0.54 |
| Sepsis | 0.11 | 0.42 |
| Other cardiovascular disorder | -1.17 | 0.29 |
| Multiple trauma | -1.11 | 0.45 |
| GI bleeding | -0.42 | 0.44 |
| GI surgery for neoplasm | -1.76 | 0.41 |
| GI perforation/obstruction | -0.97 | 0.33 |
| Other gastrointestinal disorder | -0.96 | 0.31 |


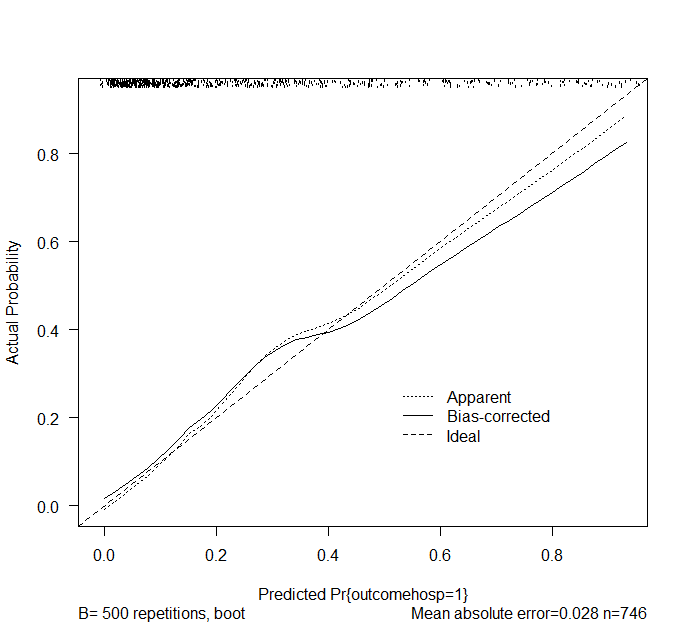

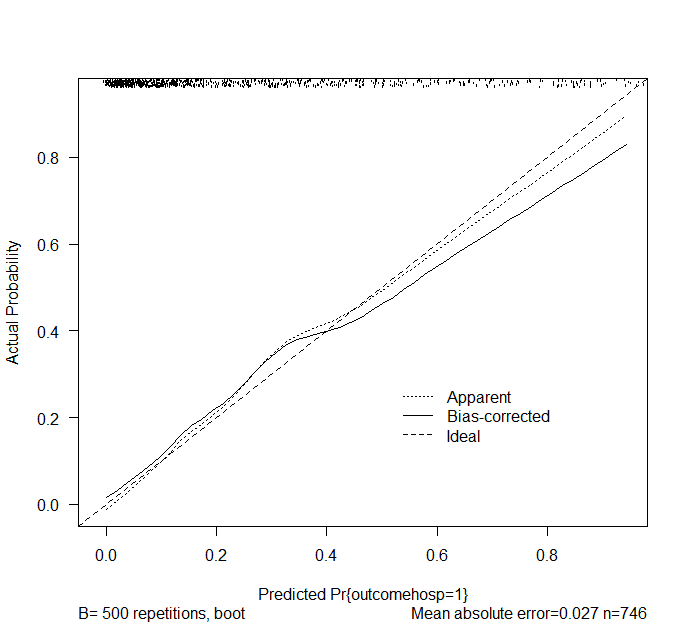


**Figure S1: Calibration plot for predicted vs actual probability of hospital mortality.** A: APACHE model, B: APACHE + TnI


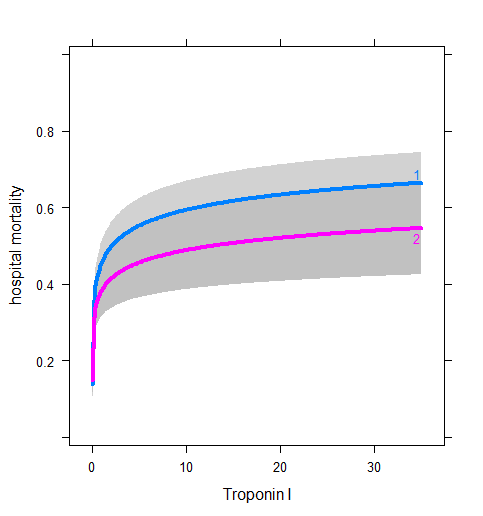


**Figure S2: Association of Troponin I (mg/l) with hospital mortality with interaction term for sex.** 1=Male, 2=Female. Hospital mortality appears higher in the male group, however, the 95% confidence intervals overlap, and if an interaction was present, the gradient of the two lines would differ (Interaction term for sex p=0.181).

**R code:**

# Multiple Fractional Polynomial Model ------------------------------------

library(mfp)

fit <- mfp(outcomehosp ~ fp(tn1) + fp(apscore_ad), data=tn24, family = binomial())

print(fit)

an <- anova(fit)

# logistic regression -----------------------------------------------------

library(rms)

d <- tn24

dd <- datadist (d); options (datadist = "dd")

# univariate tn1

f <- lrm(outcomehosp ~ I(log2(tn1+0.001)), data = tn24, x=TRUE, y=TRUE)

plot(Predict(f, tn1, fun=plogis),

xlab = "Troponin I",

ylab="hospital mortality", ylim=c(-0.02, 1.02), lwd=4)

# multivariate model

f1 <- lrm(outcomehosp ~ I(log2(tn1+0.001)) + apscore_ad + emsurg + corrapiidiag,

data = d, x=TRUE, y=TRUE)

plot(Predict(f1, tn1, fun=plogis),

ylab="Pr[Hospital Mortality]", ylim=c(-0.02, 1.02),

xlab = "Troponin I", lwd=4, cex.lab=14)

my.calib <- rms::calibrate(f1, method="boot", B=500) # model calibration

plot(my.calib, las=1)

##stratify by gender/interaction term

f2 <- lrm(outcomehosp ~ I(log2(tn1+0.001)) + sex + sex*I(log2(tn1+0.001)), data = d)

an <- anova(f2)

an

summary(f2,tn1=c(1,2))

plot(Predict(f2, tn1, "sex", fun=plogis),

xlab = "Troponin I",

ylab="hospital mortality", ylim=c(-0.02, 1.02), lwd=4)

##troponin as categorical variable

f3 <- lrm(outcomehosp ~ apscore_ad + tropcat + emsurg + corrapiidiag, data = d)

# correlation -------------------------------------------------------------

c1 <- lm(lntn1 ~ emsurg, df)

summary(c1)

sqrt(0.0095)

c2 <- lm(lntn1 ~ corrapiidiag, df)

summary(c2)

sqrt(0.1578)

c3 <- lm(lntn1 ~ apage, df)

summary(c3)

sqrt(0.02765)

c4 <- lm(df$lntn1 ~ df$aps_ad)

summary(c4)

sqrt(0.1533)

c5 <- lm(df$lntn1 ~ df$che)

summary(c5)

sqrt(0.01269)

# ROC curves --------------------------------------------------------------

library(ROCR)

#model for apiiscore, apscore_ad, tropcat, tn1 etc and ROC in derivation cohort

m1.logit <- glm (outcomehosp ~ apscore_ad + emsurg + corrapiidiag,

family = binomial(link = "logit"), data = tn24)

m2.logit <- glm (outcomehosp ~ I(log2(tn1+0.001)) + apscore_ad + emsurg + corrapiidiag,

family = binomial(link = "logit"), data = tn24)

m3.logit <- glm (outcomehosp ~ I(log2(tn1+0.001)),

family = binomial(link = "logit"), data = tn24)

summary(m2.logit)

#predicted probabilities for glasgow data

predpr1 <- predict(m1.logit, data=tn24, type = "response")

m1.scores <- prediction(predpr1, tn24$outcomehosp)

predpr2 <- predict(m2.logit, data=tn24, type = "response")

m2.scores <- prediction(predpr2, tn24$outcomehosp)

predpr3 <- predict(m3.logit, data=tn24, type = "response")

m3.scores <- prediction(predpr3, tn24$outcomehosp)

predpr4 <- predict(m4.logit, data=tn24, type = "response")

m4.scores <- prediction(predpr4, tn24$outcomehosp)

# predicted probabilities for new data

predpr1 <- predict(m1.logit, newdata=tnost, type = "response")

m1.scores <- prediction(predpr1, tnost$outcomehosp)

predpr2 <- predict(m2.logit, newdata=tnost, type = "response")

m2.scores <- prediction(predpr2, tnost$outcomehosp)

predpr3 <- predict(m3.logit, newdata=tnost, type = "response")

m3.scores <- prediction(predpr3, tnost$outcomehosp)

##ROC curve with AUC

roc.perf1 <- performance(m1.scores, measure = "tpr", x.measure = "fpr")

roc.perf2 <- performance(m2.scores, measure = "tpr", x.measure = "fpr")

roc.perf3 <- performance(m3.scores, measure = "tpr", x.measure = "fpr")

plot(roc.perf1,lwd=4, col = as.list(1:10))

plot(roc.perf2, add = TRUE, lty = 2, col = "red", lwd=4)

plot(roc.perf3, add = TRUE, lty = 8, col = "green", lwd=4)

abline(a=0, b= 1, lty = 8, col = "grey")

auc1 <- performance(m1.scores,"auc")

auc2 <- performance(m2.scores,"auc")

auc3 <- performance(m3.scores, "auc")

rocobj<- roc(tnost$outcomehosp, predpr1)

ci.auc(rocobj)

rocobj1 <- roc(tnost$outcomehosp, predpr2)

ci.auc(rocobj1)

roc.test(rocobj,rocobj1)

##Multiple Imputation for routine subgroup

library(mice)

library(VIM)

imp <- mice(dfrout, method="pmm", maxit=50, seed=500, print=FALSE)

m3 <- with(imp,glm(outcomehosp ~ I(log2(tn1+0.001)) + apscore_ad + emsurg + I(as.factor(corrapiidiag)),

family=binomial()))

m3

pooled <- pool(m3)

round(summary(pooled),2)
